# Sex differences in bladder cancer pathology and survival: analysis of a population-based cancer registry

**DOI:** 10.1002/cam4.379

**Published:** 2014-12-23

**Authors:** Masayoshi Zaitsu, Satoshi Toyokawa, Akiko Tonooka, Fumiaki Nakamura, Takumi Takeuchi, Yukio Homma, Yasuki Kobayashi

**Affiliations:** 1 Department of Public Health, The University of TokyoTokyo, Japan; 2 Department of Urology, Kanto Rosai HospitalKawasaki, Japan; 3 Department of Urology, The University of TokyoTokyo, Japan; 4 Department of Pathology, Kanto Rosai HospitalKawasaki, Japan

**Keywords:** Bladder cancer, epidemiological characteristic, Japanese, pathology, population-based, sex difference, survival

## Abstract

Sex differences in bladder cancer pathology and epidemiology have been the focus of recent research. We investigated the epidemiological characteristics and compared bladder cancer pathology and survival between men and women in Japan. A total of 13,184 patients with primary bladder cancer diagnosed from 1954 to 2010 were identified in a large-scale cancer registry database in Kanagawa Prefecture. Using this database, we compared the odds ratios (ORs) for nonurothelial carcinoma (non-UC) using a multiple logistic regression model adjusted for age and diagnosis periods. We also compared hazard ratios (HRs) for overall death and cancer-specific death using a Cox proportional hazards model adjusted for non-UC, age, and diagnosis period. The proportion of non-UC was significantly higher in female compared with male patients (OR = 2.14, 95% confidence interval [CI]: 1.81–2.52). Furthermore, survival was significantly poorer in female patients than in male patients after adjusting for UC or non-UC (HR for overall death = 1.15, 95% CI: 1.06–1.23; HR for cancer-specific death = 1.39, 95% CI: 1.28–1.52). Sex differences exist in the epidemiological characteristics of bladder cancer in Japan, with female patients having less favorable pathology and poorer survival compared with male patients.

## Introduction

Sex differences in bladder cancer epidemiology have been a focus of recent research. Female patients appear to have more unfavorable pathology and poorer survival compared with male patients. Sex differences in bladder cancer mortality have often been reported 1–3, which may be partially explained by the higher proportion of unfavorable pathology in female patients. Nonurothelial carcinoma (non-UC) is a rare form of bladder cancer with aggressive behavior and poor prognosis 4–6. Large, population-based studies using data from Surveillance, Epidemiology, and End Results (SEER) and the Netherlands Cancer Registry showed that survival was poorer in patients with non-UC, compared with those with UC 7,8. In addition, racial differences in the percentage of non-UC bladder cancer have also been reported between Caucasians and African Americans (female Caucasians 4.3% vs. female African Americans 10.5%, male Caucasians 2.3% vs. male African Americans 5.9%), while survival was poorer in females after adjusting for pathology 9.

However, to the best of our knowledge, there is currently little information regarding the existence of sex differences in bladder cancer pathology and mortality in Asians, except for one study in Japan 10. Furthermore, it investigated only patients who underwent radical cystectomy, and was unable to show any sex difference in mortality irrespective of pathology 10. We therefore conducted a whole patient survey using a large, population-based, cancer registry database of over 20,000 patients with bladder cancer in Japan. We investigated sex differences in bladder cancer pathology and mortality and determined if survival remained poorer in female than in male patients, even after adjusting for non-UC.

## Methods

### Data source

Kanagawa Cancer Registry, which covers approximately 7% of the Japanese population, is a large, population-based, cancer registry in Kanagawa Prefecture and part of the Japanese Association of Cancer Registries 11. Kanagawa Prefecture is a metropolitan prefecture located close to Tokyo. It has a population of over nine million, the second largest in Japan, and contains urbanized and rural areas. Data are registered from diagnosing institutions both inside and outside the prefecture if the patient is a resident of the prefecture. The registry database is updated with population registers and death certificates. There is no overlap for the same patient for the same disease in the database.

The database contains the following information: (1) personal identification code; (2) method of registry entry (registry system, population register, death certificate); (3) diagnosing institution; (4) sex; (5) date of birth; (6) date at diagnosis; (7) local-government code for the patient's home address; (8) International Classification of Diseases, 10th Revision (ICD-10) code for disease name; (9) International Classification of Diseases for Oncology, Third Edition (ICD-O-3) code for pathology; (10) initial or recurrent tumor; (11) date of death; (12) cause of death; (13) date of last follow-up; and (14) TNM classification according to the UICC TNM Classification of Malignant Tumours 12 and pathological grade of ICD-O-3 among patients diagnosed after 2005. We obtained the data in an anonymous format, under a research agreement with Kanagawa Cancer Registry.

Details of the database have been described previously 11. In brief (1) all information is gathered by well-trained tumor registrars certificated by the training program of Japanese Association of Cancer Registries, whose program is permitted by the SEER program; (2) follow-up information is automatically updated annually from population registers and death certificates; (3) pathological information is coded by ICD-O-3; and (4) previous versions of pathological codes have been transformed to the latest versions through standardized regulation consistent with changes in bladder cancer coding practice. Death Certificate Only (DCO) indicates patients who were only registered with “bladder cancer” according to the death certificate, with no pathological information. We used a proportion of patients with DCO as an indicator of the precision of the database, with a cut-off value of 20% 5. The proportion of DCO in our study was 16.2%, indicating that the quality of our database was adequate.

### Study subjects

The inclusion criterion was patients with bladder cancer (C67 in ICD-10) in the Kanagawa Cancer Registry. Exclusion criteria were as follows: (1) missing pathology (UC or non-UC), (2) vague pathology only identified as malignant tumor, carcinoma, or sarcoma, (3) benign tumor, (4) metastatic tumor from other sites, (5) recurrent tumor, (6) patients living outside Kanagawa Prefecture at diagnosis, (7) missing age, and (8) missing sex data.

### Variables

#### Age

Age at diagnosis was calculated and patients were classified as <65, ≥65 and <75, and ≥75 years old.

#### Pathology

Tumor pathology was divided into seven groups based on the ICD-O-3 code, according to the World Health Organization International Histological Classification of Tumours and the International Agency for Research on Cancer, with some modifications (Table2) 4,5: (1) UC, (2) squamous cell carcinoma (SCC), (3) adenocarcinoma (AC), (4) neuroendocrine tumor (NET), (5) undifferentiated carcinoma (undiff), (6) sarcoma, and (7) others. SCC, AC, NET, undiff, sarcoma, and others were all classified as non-UC.

#### Period, pathological stage, and pathological grade

On the basis of the definitions of the UICC TNM staging system 12 and previous studies corresponding to the American Joint Committee on Cancer staging system 13, we divided the date at diagnosis into: Period 0, 1954–1992; Period 1, 1993–2002; and Period 2, 2003–2010. We divided the pathological stages into: early (0is, 0a, I) and late (II–IV) stages. We divided the pathological grades into low grade (1, 2) and high grade (3, 4).

#### Observation period

The observation period was a 5-year, right-censored period. Causes of death were divided into overall death and cancer-specific death.

### Statistical methods

The primary aim of the study was to analyze the pathology and survival of patients with bladder cancer, and to detect any sex differences. We used *t*-tests, χ^2^ tests, and Fisher's exact tests to compare baseline characteristics between two groups. Proportions of non-UC patients were compared using χ^2^ and Cochran–Mantel–Haenszel tests stratified by each period. Adjusted odds ratios (ORs) for non-UC were estimated using a multiple logistic regression model adjusted for age and periods. We estimated the 5-year overall survival (5y-OS) and 5-year cancer-specific survival (5y-CSS) using the Kaplan–Meier method. Cox proportional hazards model adjusted for non-UC, age, and period was used to estimate adjusted hazard ratios (HRs) for overall death and cancer-specific death. We also estimated HRs adjusted for non-UC, age, pathological stage, and pathological grade among the patients in Period 2 because TNM classification information was available after 2005.

All *P*-values were two-sided, and *P* < 0.05 was considered statistically significant. Data were analyzed using STATA/MP13.0 (Stata-Corp LP, College Station, TX).

### Ethical considerations

The study was approved by the Institutional Review Boards of The University of Tokyo and Kanto Rosai Hospital.

## Results

We initially included 22,388 patients diagnosed from June 15, 1954 to November 22, 2010. We then excluded: (1) 8723 patients based on lack of precise pathology, (2) 475 patients with recurrent tumors, (3) two patients who lived outside Kanagawa Prefecture at diagnosis, and (4) four patients with missing age data. A total of 13,184 patients with primary bladder cancer diagnosed from June 15, 1954 to November 22, 2010 thus comprised the study subjects. The baseline characteristics of the 13,184 patients are shown in Table1. The proportion of female patients was 21.7% (2857 of 13,184). The mean ages (SD) of the female and male patients were 70 (±12.9) years and 68 (±11.8) years, respectively (*P *<* *0.001).

**Table 1:** Baseline characteristics of bladder cancer patients

Characteristic^1^	*N* (%)^2^		*P*-value^3^
	Female	Male	
Total	2857 (100)	10,327 (100)	
Age (y)			
<65	874 (30.6)	3739 (36.2)	<0.001
65–74	873 (30.6)	3471 (33.6)	
≥75	1110 (38.8)	3117 (30.2)	
Period^4^			
0	849 (29.7)	2751 (26.6)	0.002
1	1040 (36.4)	3780 (36.6)	
2	968 (33.9)	3796 (36.8)	

1 Data for 13,184 patients with complete information on sex, pathology, age, and period.

2 Because of rounding, percentages may not total 100.

3 *P*-values for χ^2^ tests comparing female and male patients.

4 Period: Total, 1954–2010; Period 0, 1954–1992; Period 1, 1993–2002; Period 2, 2003–2010.

### Sample size

The sample size for the analysis of pathology was large enough to show a 4% difference in the proportion of non-UC (female patients, 8%, male patients, 4%, *α* = 0.05, *β* = 0.2). The sample size for the analysis of survival was large enough to show a 5% difference in survival rate (female patients, 50%, male patients, 55%, *α* = 0.05, *β* = 0.2). The proportion of DCO was 16.2%.

### Pathology

The pathological distribution of the 13,184 patients is shown in Table2. The proportion of female patients with non-UC was significantly higher than the proportion of male patients. The overall proportions of non-UC female and male patients were 8.2% (234/2857) and 4.0% (414/10,327) (*P *<* *0.001), respectively. In addition, the proportions of urothelial carcinoma in situ (CIS) in female and male patients were 11.2% and 9.7% in Period 2, respectively.

**Table 2:** Distribution and definition of bladder cancer pathologies

Characteristics^1^	*N* (%)^2^		*P*-value^3^^,^^4^
	Female	Male	
*Overall*^5^			
UC	2623 (91.8)	9913 (96.0)	<0.001
Non-UC	234 (8.2)	414 (4.0)	
SCC	103 (3.6)	146 (1.4)	
AC	90 (3.2)	205 (2.0)	
NET	11 (0.4)	18 (0.2)	
Undiff	7 (0.2)	25 (0.2)	
Sarcoma	18 (0.6)	14 (0.1)	
Others	5 (0.2)	6 (0.1)	
*Period 0*^5^			
UC	755 (88.9)	2617 (95.1)	<0.001
Non-UC	94 (11.1)	134 (4.9)	
SCC	46 (5.4)	51 (1.8)	
AC	33 (3.9)	59 (2.1)	
NET	1 (0.1)	1 (<0.1)	
Undiff	4 (0.5)	10 (0.4)	
Sarcoma	9 (1.1)	10 (0.4)	
Others	1 (0.1)	3 (0.1)	
*Period 1*^5^			
UC	968 (93.1)	3666 (97.0)	<0.001
Non-UC	72 (6.9)	114 (3.0)	
SCC	27 (2.6)	39 (1.0)	
AC	31 (3.0)	58 (1.5)	
NET	5 (0.5)	7 (0.2)	
Undiff	2 (0.2)	7 (0.2)	
Sarcoma	4 (0.4)	1 (<0.1)	
Others	3 (0.3)	2 (<0.1)	
*Period 2*^5^			
UC	900 (93.0)	3630 (95.6)	<0.001
Non-UC	68 (7.0)	166 (4.4)	
SCC	30 (3.1)	56 (1.5)	
AC	26 (2.7)	88 (2.3)	
NET	5 (0.5)	10 (0.3)	
Undiff	1 (0.1)	8 (0.2)	
Sarcoma	5 (0.5)	3 (<0.1)	
Others	1 (0.1)	1 (<0.1)	

Characteristics	Definition by ICD-O-3^6^
UC	8120–8131 (including CIS, 8120/2), 8050
Non-UC	
SCC	8051–8078, 8083–8084
AC	8140–8231, 8250–8384, 8440–8490, 8550–8551, 8570–8576
NET	8013, 8240–8249, 8041–8045, 8680–8700
Undiff	8020–8021
Sarcoma	8801–8811, 8830, 8840–8921, 8930–8991, 9040–9044, 9120–9133, 9150, 9540–9581
Others	The others

UC, urothelial carcinoma; CIS, urothelial carcinoma in situ; SCC, squamous cell carcinoma; AC, adenocarcinoma; NET, neuroendocrine tumor; Undiff, undifferentiated carcinoma; non-UC, nonurothelial carcinoma; ICD-O-3, International Classification of Diseases for Oncology, Third Edition. Non-UC includes SCC, AC, NET, Undiff, Sarcoma, and Others.

1 Data for 13,184 patients with complete information on sex, pathology, age, and period.

2 Because of rounding, percentages may not total 100.

3 *P*-values for Fisher's exact tests comparing female and male patients.

4 All the *P*-values for χ^2^ tests comparing the proportions of non-UC female and male patients in Periods 0, 1, and 2 were <0.01.

5 Period: Total, 1954–2010; Period 0, 1954–1992; Period 1, 1993–2002; Period 2, 2003–2010.

6 Proportions of CIS in female and male patients: Period 0, 0.2% (2/849) and 0.2% (4/2751) (*P* = 0.63); Period 1, 0.5% (5/1040) and 0.5% (20/3780) (*P* > 0.99); Period 2, 11.2% (108/968) and 9.7% (369/3796) (*P* = 0.19).

The model fit was good, according to multivariate analysis (Hosmer–Lemeshow χ^2^ = 15.1, *P *=* *0.06). The ORs for non-UC were: (1) female, 2.14 (1.81–2.52), (2) age ≥65 and <75 years, 0.90 (0.75–1.10); age ≥75 years, 0.90 (0.74–1.09), (3) Period 0, 1.65 (1.35–2.01); Period 2, 1.31 (1.08–1.60). The proportion of non-UC was significantly higher in female than in male patients.

### Survival

**Figure 1:**
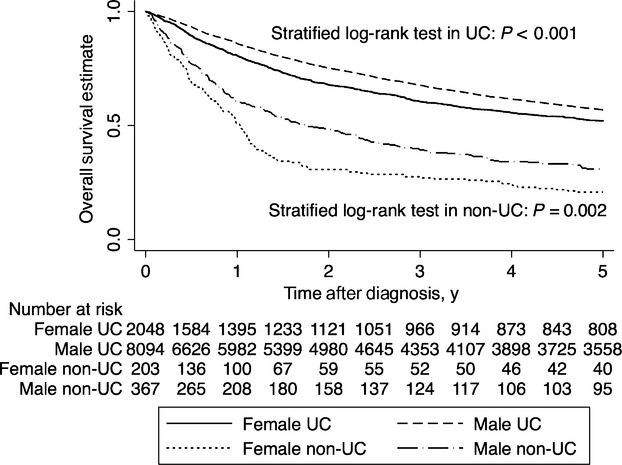
Kaplan–Meier survival estimate curves for overall survival in male and female patients with bladder cancer. Survival was estimated using the Kaplan–Meier method in 10,712 patients with complete information on sex, pathology, age, period, and observation period, with right censoring at the 5-year point. *P*-values are for stratified log-rank test with diagnosis periods. UC, urothelial carcinoma; non-UC, nonurothelial carcinoma.

**Figure 2:**
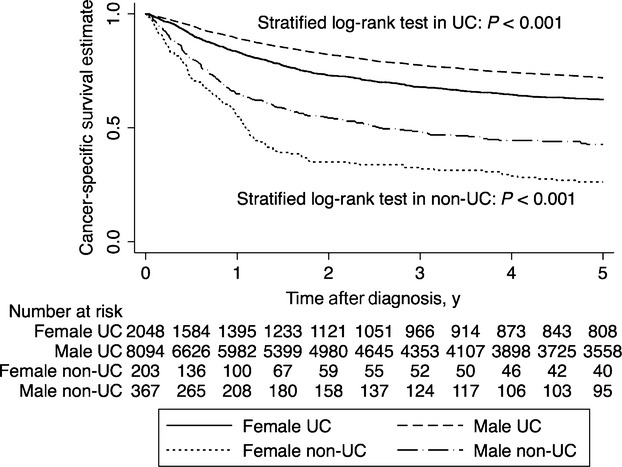
Kaplan–Meier survival estimate curves for cancer-specific survival in male and female patients with bladder cancer. Survival was estimated using the Kaplan–Meier method in 10,712 patients with complete information on sex, pathology, age, period, and observation period, with right censoring at the 5-year point. *P*-values are for stratified log-rank test with diagnosis periods. UC, urothelial carcinoma; non-UC, nonurothelial carcinoma.

We excluded 2472 of the 13,184 eligible patients because of missing observation periods because of a registry time lag, leaving 10,712 patients for analysis. The Kaplan–Meier survival estimate curves for overall death and for cancer-specific death are shown in Figures1 and 2, respectively. The 5y-OS and 5y-CSS by pathology are shown in Table3. The 5y-OS and 5y-CSS were significantly poorer in female patients compared with male patients. The 5y-OS in female and male patients were 0.49 (0.47–0.51) and 0.56 (0.54–0.57) (*P *<* *0.001); the 5y-CSS was 0.59 (0.57–0.61) and 0.71 (0.70–0.72) (*P *<* *0.001), respectively. Regardless of pathological types, the 5y-OS and 5y-CSS were poorer in female than in male patients.

**Table 3:** Five-year overall and cancer-specific survival estimates according to bladder cancer pathology

Characteristic^1^	5y-OS (95% CI)		*P*-value^2^	5y-CSS (95% CI)		*P*-value^2^
	Female	Male		Female	Male	
Overall^3^						
Total	0.49 (0.47–0.51)	0.56 (0.54–0.57)	<0.001	0.59 (0.57–0.61)	0.71 (0.70–0.72)	<0.001
UC	0.52 (0.50–0.54)	0.57 (0.56–0.58)	<0.001	0.62 (0.60–0.65)	0.72 (0.71–0.73)	<0.001
Non-UC	0.21 (0.15–0.27)	0.31 (0.26–0.36)	0.002	0.26 (0.20–0.33)	0.43 (0.37–0.48)	<0.001
Period 0^3^						
Total	0.49 (0.45–0.53)	0.56 (0.54–0.58)	<0.001	0.56 (0.52–0.59)	0.66 (0.64–0.68)	<0.001
UC	0.53 (0.49–0.57)	0.57 (0.55–0.59)	0.047	0.60 (0.56–0.64)	0.67 (0.65–0.70)	0.001
Non-UC	0.16 (0.09–0.25)	0.32 (0.23–0.40)	0.01	0.20 (0.12–0.30)	0.36 (0.27–0.45)	0.02
Period 1^3^						
Total	0.48 (0.44–0.51)	0.56 (0.54–0.58)	<0.001	0.58 (0.54–0.62)	0.72 (0.70–0.74)	<0.001
UC	0.50 (0.46–0.53)	0.57 (0.55–0.59)	<0.001	0.61 (0.57–0.64)	0.73 (0.71–0.75)	<0.001
Non-UC	0.24 (0.14–0.35)	0.27 (0.19–0.37)	0.40	0.31 (0.19–0.43)	0.40 (0.29–0.50)	0.15
Period 2^3^						
Total	0.51 (0.46–0.55)	0.54 (0.52–0.56)	0.003	0.64 (0.59–0.68)	0.73 (0.71–0.75)	<0.001
UC	0.54 (0.49–0.58)	0.56 (0.53–0.58)	0.07	0.67 (0.62–0.71)	0.75 (0.73–0.77)	<0.001
Non-UC	0.24 (0.14–0.35)	0.32 (0.23–0.40)	0.04	0.31 (0.19–0.44)	0.52 (0.42–0.61)	0.007

NA, not available; UC, urothelial carcinoma; SCC, squamous cell carcinoma; AC, adenocarcinoma; NET, neuroendocrine tumor; Undiff, undifferentiated carcinoma; non-UC, nonurothelial carcinoma; 5y-OS, 5-year overall survival estimate; 5y-CSS, 5-year cancer-specific survival estimate. Non-UC includes SCC, AC, NET, Undiff, Sarcoma, and Others.

1 Data analyzed by Kaplan–Meier method in 10,712 patients with complete information on sex, pathology, age, period, and observation period.

2 *P*-values for log-rank tests in each period and stratified log-rank tests over all periods, comparing female and male patients with each pathology.

3 Period: Total, 1954–2010; Period 0, 1954–1992; Period 1, 1993–2002; Period 2, 2003–2010.

The HRs adjusted for available confounders for overall death and cancer-specific death are shown in Table4. According to multivariate analysis, the proportional hazards assumption checked with the log–log plot of survival indicated a good model fit (data not shown). Interestingly, both overall survival and cancer-specific survival were significantly poorer in female compared with male patients (HR for overall death = 1.15 [1.06–1.23]; HR for cancer-specific death = 1.39 [1.28–1.52]). Female adjusted HRs by age were similar (Table5).

**Table 4:** Hazard ratios for overall death and cancer-specific death adjusted for available confounders

Characteristic	Hazard ratio (95% CI)^1^	
	Overall death	Cancer-specific death
Sex		
Male	1.00 (reference)	1.00 (reference)
Female	1.15 (1.06–1.23)	1.39 (1.28–1.52)
Pathology		
UC	1.00 (reference)	1.00 (reference)
Non-UC	2.68 (2.41–2.97)	3.13 (2.78–3.52)
Age (y)		
<65	1.00 (reference)	1.00 (reference)
≥65 and <75	1.41 (1.29–1.54)	1.21 (1.09–1.34)
≥75	2.50 (2.31–2.72)	1.93 (1.75–2.12)
Period^2^		
Period 0	1.07 (0.99–1.15)	1.26 (1.16–1.38)
Period 1	1.00 (reference)	1.00 (reference)
Period 2	0.97 (0.91–1.05)	0.87 (0.79–0.96)

UC, urothelial carcinoma; SCC, squamous cell carcinoma; AC, adenocarcinoma; NET, neuroendocrine tumor; Undiff, undifferentiated carcinoma; non-UC, nonurothelial carcinoma. Non-UC includes SCC, AC, NET, Undiff, Sarcoma, and Others.

1 Data analyzed by Cox proportional hazards model between the sexes adjusted for non-UC, age and Period in 10,712 patients with complete information on sex, pathology, age, period, and observation period.

2 Period: Total, 1954–2010; Period 0, 1954–1992; Period 1, 1993–2002; Period 2, 2003–2010.

**Table 5:** Hazard ratios for overall death and cancer-specific death in female patients compared with male patients by age.^1^^,^^2^

Characteristic	Female hazard ratio (95% CI)	
	Overall death	Cancer-specific death
Age (y)		
<65	1.15 (0.96–1.36)	1.22 (1.01–1.48)
≥65 and <75	1.06 (0.92–1.22)	1.23 (1.05–1.45)
≥75	1.18 (1.07–1.30)	1.56 (1.38–1.76)

1 Data analyzed by Cox proportional hazards model between the sexes adjusted for non-UC and Period in 10,712 patients with complete information on sex, pathology, age, period, and observation period.

2 Period: Total, 1954–2010; Period 0, 1954–1992; Period 1, 1993–2002; Period 2, 2003–2010.

### Survival in patients with pathological stage and pathological grade in Period 2

Of the patients in Period 2 (4764/13,184), we excluded 3706 patients with missing pathological stage, 323 patients with missing pathological grade, and 36 patients with missing survival-period data, because these data were available for most of this most-recent cohort 14. The remaining 699 patients were the subjects for analysis.

HR adjusted for non-UC and age, and HR adjusted for non-UC, age, pathological stage, and pathological grade in female compared with male patients were as follows: HR for overall death = 1.52 (1.09–2.12) and 1.52 (1.09–2.13), and HR for cancer-specific death = 1.69 (1.13–2.52) and 1.71 (1.14–2.56), respectively.

## Discussion

The results of this study demonstrated the existence of a sex difference in the epidemiology of bladder cancer in Japanese patients. Compared with male patients, the proportion of non-UC was twice as high in female patients (OR = 2.14, 1.81–2.52), while survival was poorer in females after adjusting for pathology, age, and period (HRs for overall and cancer-specific deaths = 1.15 [1.06–1.23] and 1.39 [1.28–1.52], respectively). It was still poorer after additional adjustment for pathological stage and pathological grade (HRs for overall and cancer-specific deaths = 1.52 [1.09–2.13] and 1.71 [1.14–2.56], respectively).

We limited the study subjects to those with complete information for each analysis, because missing information would have introduced a random element. The random missing information in large-scale samples would have statistically little impact on the point estimation because of the central limit theorem. Missing information would be associated with the requirement for medical institutions to preserve medical records for 5 years in Japan; however, this would have little impact on the difference between the sexes.

In our study population, the number of female patients with bladder cancer was around a quarter the number of males, and the adjusted female HR for cancer-specific death compared with male patients was 1.39, which is similar to those reported in Caucasians and African Americans 9. The percentages of non-UC were 8.2% in female patients 4.0% in male patients over the whole study period and all ages, which were higher than those in Caucasians and lower than those in African Americans 9, suggesting the existence of a racial difference in bladder cancer cell type. The percentage of non-UC in female patients decreased by period (Period 0: 11.1%, Period 1: 6.9%, Period 2: 7.0%). However, the relevance of diagnostic factors to this temporal trend is unclear, given that no similar trend was observed in male patients.

Sex differences in bladder cancer epidemiology may be explained by biological, past-historical, and lifestyle standpoints. First, sex differences in the dominant hormones and in vascularity around the bladder may be a relevant factor. From a hormonal perspective, the incidence of bladder cancer was shown to be higher in postmenopausal than in premenopausal women 15, and aggressive bladder cancers expressed high levels of estrogen receptor-*β* and few androgen receptors 16–18. From a vascular perspective, bladder cancers with high vascularity have been associated with poorer survival than those with low vascularity 19,20; similarly, high expression of vascular endothelial growth factor (VEGF) was associated with poorer survival than low expression of VEGF 21. Second, a difference in the incidence of cystitis between the sexes may also help to explain the difference in bladder cancer epidemiology. Compared with patients who had never experienced cystitis, patients who had experienced at least three episodes of cystitis had increased risks of whole and SCC bladder cancers 22. Finally, sex differences in lifestyle-related risk factors for bladder cancer, such as smoking 23–25, occupational exposure to particular aromatic amines 26,27, nuclear pollution 28,29, economic status 3, and potential delay in diagnosis in female patients 30 could also explain the sex difference.

Our study had several limitations, including some selection biases, and the fact that the pathological coding practices for bladder cancer changed over the study period. Furthermore, we had no information on individual patient attributes, such as history of cystitis, smoking, occupational exposure, environmental pollution, economic status, and treatment. (1) Selection bias might have affected the external validity of this observational study; however, the quality of the prefecture-wide survey, with a DCO percentage of 16.2%, suggested that the precision of the estimates was high and selection bias could be ignored. (2) The proportion of CIS was around 10% in the last decade, which was much higher than in preceding decades; however, this trend is probably associated with changes in coding practices. Coding changes would influence the conclusions in the same direction in both sexes, suggesting that any impact of coding changes could be ignored after adjusting for periods. (3) Smoking is related to poor survival in bladder cancer 23. The Vital Statistics of Japan (www.e-stat.go.jp) show that the percentage of female smokers is about one-third that of male smokers, suggesting that male patients should have poorer survival than female patients. We are currently collecting data on the smoking status of patients in Kanagawa Cancer Registry, and these results will be incorporated in a future study. (4) Lack of socioeconomic information may represent a weakness of our study because of the impact of socioeconomic status on mortality; however, the effect on sex differences would likely be negligible. Japan has a universal public healthcare system that extensively covers standardized and recommended examinations and treatments within cancer guidelines, regardless of the patient's socioeconomic status. Female and male patients would thus have equal chances to receive examinations and treatment, regardless of their socioeconomic status. The lack of socioeconomic information should therefore not weaken the conclusions. However, further studies are needed to address these limitations.

The strength of this study was the fact that it is the first population-based study to demonstrate sex differences in both pathology and mortality of bladder cancer in Japan. Furthermore, survival was significantly poorer in female patients than male patients after adjusting for either UC or non-UC. The results thus represent an important step in reconsidering the treatment strategy for this disease. Although guideline-based strategies for bladder cancer treatment have been recommended for many years, they have not addressed the survival difference between the sexes. The novel Osaka Medical College regimen 31, which is based on a new concept outside the guidelines, suggests that the combination of radiotherapy, extensive high-dose chemotherapy with balloon-occluded arterial infusion, and hemodialysis may have a curative effect on advanced bladder cancer in both female and male patients. Our results suggest that the main strategy for treating bladder cancer might take into account the sex of the patient. In summary, there are sex differences in the epidemiological characteristics of bladder cancer, and female patients have unfavorable pathology and poorer survival compared with male patients. Further studies are needed to confirm female sex as an independent risk factor for these unfavorable characteristics.

## References

[b1] Micheli A, Mariotto A, Giorgi Rossi A, Gatta G, Muti P (1998). The prognostic role of gender in survival of adult cancer patients. EUROCARE working group. Eur. J. Cancer.

[b2] Madeb R, Messing EM (2004). Gender, racial and age differences in bladder cancer incidence and mortality. Urol. Oncol.

[b3] Kirkali Z, Chan T, Manoharan M, Algaba F, Busch C, Cheng L (2005). Bladder cancer: epidemiology, staging and grading, and diagnosis. Urology.

[b4] Eble JN, Sauter G, Epstein JI, Sesterhenn IA (2004). World Health Organization classification of tumours. Pathology and genetics of tumours of the urinary system and male genital organs.

[b5] Curado MP, Edwards B, Shin HR, Storm H, Ferlay J, Heanue M (2007). Cancer incidence in five continents.

[b6] Black PC, Brown GA, Dinney CPN (2009). The impact of variant histology on the outcome of bladder cancer treated with curative intent. Urol. Oncol.

[b7] Wright JL, Black PC, Brown GA, Porter MP, Kamat AM, Dinney CP (2007). Differences in survival among patients with sarcomatoid carcinoma, carcinosarcoma and urothelial carcinoma of the bladder. J. Urol.

[b8] Ploeg M, Aben KK, Hulsbergen-van de Kaa CA, Schoenberg MP, Witjes JA, Kiemeney LA (2010). Clinical epidemiology of nonurothelial bladder cancer: analysis of The Netherlands Cancer Registry. J. Urol.

[b9] Scosyrev E, Noyes K, Feng C, Messing E (2009). Sex and racial differences in bladder cancer presentation and mortality in the US. Cancer.

[b10] Nishiyama H, Habuchi T, Watanabe J, Teramukai S, Tada H, Ono Y (2004). Clinical outcome of a large-scale multi-institutional retrospective study for locally advanced bladder cancer: a survey including 1131 patients treated during 1990–2000 in Japan. Eur. Urol.

[b11] Okamoto N (2008). A history of the cancer registration system in Japan. Int. J. Clin. Oncol.

[b12] Sobin LH, Wittekind Ch (2002). UICC (International Union Against Cancer) TNM classification of malignant tumours.

[b13] David KA, Mallin K, Milowsky MI, Ritchey J, Carroll PR, Nanus DM (2009). Surveillance of urothelial carcinoma: stage and grade migration, 1993–2005 and survival trends, 1993–2000. Cancer.

[b14] Higashi T, Nakamura F, Shibata A, Emori Y, Nishimoto H (2014). The national database of hospital-based cancer registries: a nationwide infrastructure to support evidence-based cancer care and cancer control policy in Japan. Jpn. J. Clin. Oncol.

[b15] McGrath M, Michaud DS, De Vivo I (2006). Hormonal and reproductive factors and the risk of bladder cancer in women. Am. J. Epidemiol.

[b16] Shen SS, Smith CL, Hsieh J, Yu J, Kim IY, Jian W (2006). Expression of estrogen receptors-α and -β in bladder cancer cell lines and human bladder tumor tissue. Cancer.

[b17] Boorjian S, Ugras S, Mongan NP, Gudas LJ, You X, Tickoo SK (2004). Androgen receptor expression is inversely correlated with pathologic tumor stage in bladder cancer. Urology.

[b18] Tuygun C, Kankaya D, Imamoglu A, Sertcelik A, Zengin K, Oktay M (2011). Sex-specific hormone receptors in urothelial carcinomas of the human urinary bladder: a comparative analysis of clinicopathological features and survival outcomes according to receptor expression. Urol. Oncol.

[b19] Bochner BH, Cote RJ, Weidner N, Groshen S, Chen S, Skinner DG (1995). Angiogenesis in bladder cancer: relationship between microvessel density and tumor prognosis. J. Natl. Cancer Inst.

[b20] Dickinson AJ, Fox SB, Persad RA, Hollyer J, Sibley GN, Harris AL (1994). Quantification of angiogenesis as an independent predictor of prognosis in invasive bladder carcinomas. Br. J. Urol.

[b21] Crew JP, O'Brien T, Bradburn M, Fuggle S, Bicknell R, Cranston D (1997). Vascular endothelial growth factor is a predictor of relapse and stage progression in superficial bladder cancer. Cancer Res.

[b22] Kantor AF, Hartge P, Hoover RN, Narayana AS, Sullivan JW, Fraumeni JF (1984). Urinary tract infection and risk of bladder cancer. Am. J. Epidemiol.

[b23] Akiba S, Hirayama T (1990). Cigarette smoking and cancer mortality risk in Japanese men and women—results from reanalysis of the six-prefecture cohort study data. Environ. Health Perspect.

[b24] Brennan P, Bogillot O, Cordier S, Greiser E, Schill W, Vineis P (2000). Cigarette smoking and bladder cancer in men: a pooled analysis of 11 case-control studies. Int. J. Cancer.

[b25] Zeegers M, Swaen G, Kant I, Goldbohm R, Van den Brandt P (2001). Occupational risk factors for male bladder cancer: results from a population based case cohort study in the Netherlands. Occup. Environ. Med.

[b26] Malker HS, McLaughlin JK, Silverman DT, Ericsson JL, Stone B, Weiner JA (1987). Occupational risks for bladder cancer among men in Sweden. Cancer Res.

[b27] Naito S, Tanaka K, Koga H, Kotoh S, Hirohata T, Kumazawa J (1995). Cancer occurrence among dyestuff workers exposed to aromatic amines. A long term follow-up study. Cancer.

[b28] Romanenko A, Morimura K, Wanibuchi H, Wei M, Zaparin W, Vinnichenko W (2003). Urinary bladder lesions induced by persistent chronic low-dose ionizing radiation. Cancer Sci.

[b29] Ozasa K, Shimizu Y, Suyama A, Kasagi F, Soda M, Grant EJ (2011). Studies of the mortality of atomic bomb survivors, report 14, 1950-2003: an overview of cancer and noncancer diseases. Radiat. Res.

[b30] Garg T, Pinheiro LC, Atoria CL, Donat SM, Weissman JS, Herr HW (2014). Gender disparities in hematuria evaluation and bladder cancer diagnosis: a population based analysis. J. Urol.

[b31] Azuma H, Inamoto T, Ibuki N, Ubai T, Kotake Y, Takahara K (2010). Novel bladder preservation therapy for locally invasive bladder cancer: combined therapy using balloon-occluded arterial infusion of anticancer agent and hemodialysis with concurrent radiation. Int. J. Oncol.

